# Comparative Analysis of Machine Learning and Numerical Modeling for Combined Heat Transfer in Polymethylmethacrylate

**DOI:** 10.3390/polym14101996

**Published:** 2022-05-13

**Authors:** Mahsa Dehghan Manshadi, Nima Alafchi, Alireza Tat, Milad Mousavi, Amirhosein Mosavi

**Affiliations:** 1 Institute of Information Society, National University of Public Service, 1083 Budapest, Hungary; mahsadehghan@email.kntu.ac.ir (M.D.M.); miladmousavi@email.kntu.ac.ir (M.M.); 2 Department of Biomedical Engineering, Payame Noor University, Tehran 19395-4697, Iran; nima.alafchi@yahoo.com; 3 Department of Industrial Engineering, Payame Noor University, Tehran 19395-4697, Iran; alirezatat1994@gmail.com; 4 John von Neumann Faculty of Informatics, Obuda University, 1034 Budapest, Hungary; 5 Institute of Information Engineering, Automation and Mathematics, Slovak University of Technology in Bratislava, 81107 Bratislava, Slovakia

**Keywords:** polymethylmethacrylate, heat transfer, heat conduction, machine learning, confusion matrix, polymer, long short-term memory, deep learning, artificial intelligence, information systems

## Abstract

This study has compared different methods to predict the simultaneous effects of conductive and radiative heat transfer in a polymethylmethacrylate (PMMA) sample. PMMA is a type of polymer utilized in various sensors and actuator devices. One-dimensional combined heat transfer is considered in numerical analysis. Computer implementation was obtained for the numerical solution of the governing equation with the implicit finite difference method in the case of discretization. Kirchhoff transformation was used to obtain data from a non-linear equation of conductive heat transfer by considering monochromatic radiation intensity and temperature conditions applied to the PMMA sample boundaries. For the deep neural network (DNN) method, the novel long short-term memory (LSTM) method was introduced to find accurate results in the least processing time compared to the numerical method. A recent study derived the combined heat transfer and temperature profiles for the PMMA sample. Furthermore, the transient temperature profile was validated by another study. A comparison proves the perfect agreement. It shows the temperature gradient in the primary positions, which provides a spectral amount of conductive heat transfer from the PMMA sample. It is more straightforward when they are compared with the novel DNN method. Results demonstrate that this artificial intelligence method is accurate and fast in predicting problems. By analyzing the results from the numerical solution, it can be understood that the conductive and radiative heat flux are similar in the case of gradient behavior, but the amount is also twice as high approximately. Hence, total heat flux has a constant value in an approximated steady-state condition. In addition to analyzing their composition, the receiver operating characteristic (ROC) curve and confusion matrix were implemented to evaluate the algorithm’s performance.

## 1. Introduction

In recent decades, communication among human beings has increased. Because of the high growth rate, this issue needs to be improved by communication systems and data transferring. The most suitable material for these applications is optical fiber, so researchers have studied the different types of composite optical fibers, especially plastic optical fiber (POF) [1,2]. Beyond these activities, the transmission medium’s production was stopped for a few years because of the lack of commercial field claims. Composite fiber has various applications, such as light transmission sensing, data transmission for optical signals, and deformation detection, which is used in solid mechanical experimental research and chemical sensing [3,4,5,6]. Their applications are critical because they have improved the reliability of electrical machines, ICs, and microelectronics. Nevertheless, this research focused on using the polymethylmethacrylate (PMMA) plastic optical fiber (PMMA-POF) for data transferring. POF is one of the polymer categories that can easily be integrated into the textile statement. One of the mechanical properties of POF is its electromagnetic radiation and it does not have any heat generation [7]. Related to the above, researchers focus on the penalties that POF may entail for the performance of the transmission. The physical characteristics of PMMA-POF are the core feature size of 1.49 and 1.59 mm. The use of silicone resin was investigated, with an approximate refractive index of 1.46 mm for fiber cladding, maintaining a high difference between core and cladding, and mechanical flexibility. Attenuation loss is approximately 1 nm at 650 nm, and the total price decreases [8].

One of the crucial factors for determining the maximum length of the fiber link is attenuation. It depends on the wavelength and is also related to its material properties. Three types of windows can be named here, related to the attenuation values. They exist around 500 nm, 570 nm, and 650 nm, starting from at least 80 dB/km. If the value ranges are in the visible wavelength, colors will show these windows, predominantly blue–green, yellow, and red. The low attenuation is related to the green and blue windows, even though there are lower values related to the yellow color, which is neglected here because of low magnitudes; still, the red one has a higher value at a higher rate of speed [9]. Furthermore, these factors can ultimately affect heat transfer. Conductive heat transfer is extensively influenced by the physical properties of a material. However, the selected material for this study is the optimum one in the case of combined heat transfer. For the PMMA-POF, the transmission length becomes limited to the range of ten or a hundred meters, which depends on the baud rate. For the diverse data communication applications over PMMA-POF, the major market is introduced as home or office networks. When the system is going to dimension and then installed, the bending loss becomes an essential parameter. For 360° bending, the different values of extra-losses are determined by the equilibrium model. It can be considered that the bending extra-attenuation is near 0.5 dB for a 10 mm bending radius of PMMA-POF material, while the extra-loss is near zero when the bending radius becomes at least 25 mm [9]. By selecting the PMMA-POF material, energy can easily be transferred by combined radiation and conduction heat transfer internally because of the high data transfer speed. Consequently, radiation’s role as the heat transfer type is more important than the conductive heat transfer [9].

Multitudes of researchers have examined the combined heat transfer effect in glass. Hemath [10] studied the fiber conductivity of reinforced composites by considering radiation. Sallam [11] studied a parameter that can affect radiation shielding parameters. Li et al. [12] conducted a thermal performance evaluation of glass windows combining silica aerogels and phase change materials for the cold climate of China. Barnoss et al. [13] analyzed transient heat transfer in a semitransparent area to predict the temperature distribution, and also they predicted conduction and radiation transient heat transfer at high temperatures [14]. Malek et al. [15] studied a transient numerical thermal radiation solution on a medium. A numerical solution of the Rosseland model for transient thermal radiation in non-grey optically thick media was obtained using enriched basis functions. Prerana and Satapathy [16,17] investigated the transient conduction and radiation problem using the finite volume method. Wakif A. analyzed the convection heat transfer on steady flow by considering the radiative effect on a horizontal sheet. O. D. Makinde [18] analyzed the mass transfer past a moving vertical porous plate with a thermal radiation effect. Moreover, Chu et al. [19] studied the combined impacts of the thermal dependence in convection heat transfer by considering radiative heat flux boundary conditions. Among all of the tools used in the industry, machine learning with artificial neural network (ANN) and Internet of Things (IoT) is a powerful modeling and forecasting tool that offers an alternative means to solve complex problems, such as predicting production capacity [20]. In addition, several studies were conducted in the case of different machine learning methods’ approximations in the heat transfer field. For example, Kwon et al. investigated the best correlation between heat transfer parameters [21]. Potočnik et al. performed a prediction of the amount of heat demand in a district heating system application. They utilized the machine learning method to introduce a novel heat transfer model for a multi-step heat demand [22]. Moreover, Zhao et al. [23] researched estimation with a machine learning method when paired with special sensors. They were successful in estimating the peak temperature and heat source localization. Ju and Shiomi [24] studied the material informatic in the case of heat transfer applications. They discuss recent progress in developing material informatics (MI) for heat transport. Their review indicates that these methods are the most beneficial for designing thermal functional materials. Moreover, Ju et al. [25] also studied the coupled machine learning and thermal transport effects for designing a material with a thermal function. They claim that there is a significant gap in the amount of data collected from numerical and experimental modeling. Thus, transfer learning plays a vital role in filling this gap. The utilized machine learning algorithm was the Bayesian optimization algorithm. Furthermore, they introduced future fields of study.

This study develops an approximation method for assessing the radiative and conductive heat transfer in a PMMA sample. A finite difference scheme is used to obtain a model of the combined heat transfer. The equations are derived for a glass optical fiber, with PMMA subjected to different boundaries. The temperature distribution and the heat fluxes of two named heat transfer types are obtained. A comparison is made with the solution of computer implementation, and the data are used in an LSTM algorithm to find the best correlation between all of the relative heat transfer parameters. One of the most significant aims of this research is to perform a comparative analysis between two different schemes in order to reduce the calculation time. Hence, the correlation matrix is utilized to find the relation between essential parameters. Then, the receiver operating characteristic (ROC) curve is utilized in order to compare different machine learning methods’ accuracy.

## 2. Materials and Methods

### 2.1. Numerical Analysis

This research focused on the measurements of plastic optical fiber for polymethylmethacrylate or so called PMMA-POF at different temperatures. In general, a plastic or glass optical fiber (GOF) consists of three main layers. The inner layer is named “core transfer data”. The next layer covers the core and should limit the light transmission in a line. These two layers are made of pure silica glass. The outer layer is considered a coating that protects the inner ones and is composed of a plastic or acrylate covering. However, for the POF, the core material is general-purpose resin, and the core covering (cladding) is made of a fluorinated polymer [26]. In addition, for PMMA-POF, two wires made of tantalum, with 25 μm diameters, are used here for experimental modeling. 

When measuring thermal conductivity, electricity is used. When there is a current in the wires for a short period (5 s), they will be heated to their transient temperature. When the PMMA-POF becomes hot, a significant internal emission will happen. This means that energy is transferred from one layer to another by the radiation effect. Thus, the transient response of the thermal effect is very different when it is combined with heat transfer [27]. For measuring the resistance of a wire, the computer program of the Wheatstone bridge is used [28]. The two equations of conductive and radiative heat transfer should simultaneously solve the case of combined heat transfer. The first significant step in any numerical analysis is to select the studied material and its properties. The selected material is PMMA and its properties are defined in Table 1.

The sample material, PMMA-POF, has time-dependent physical properties, anisotropic absorption, is a non-gray material and has emission and scattering properties [29]. 

The assumption is that the front face represents the shock temperature, and the back face is a uniform temperature. In addition, a medium boundary temperature is assumed, and the initial temperature is considered to be uniform.

For radiation analysis of the PMMA-POF wire, several considerations should be applied to theoretical modeling. Although many methods exist for analyzing radiative heat transfer, the monochromatic radiation intensity *J*_(λ (*X.μ.t*)) is obtained from the radiative heat transfer equation (RTE) [29]. Equation (1) is given for the position *X* in a *μ* direction with a λ wavelength at time *t*.

(1) $$\frac{1}{c}\frac{\partial I_{\lambda }(X,\mu ,t)}{\partial t}+\mu \frac{\partial I_{\lambda }(X,\mu ,t)}{\partial X}=+J_{\lambda }(X,\mu ,t)-\beta_{\lambda }(\mu )I_{\lambda }(X,\mu ,t)$$

By using the order of magnitude, the first term in Equation (1) can be neglected because the propagation speed *c* is higher than the other terms. Thus, Equation (2) can be written as follows:

(2) $$\mu \frac{\partial I_{\lambda }(X,\mu ,t)}{\partial X}=-\beta_{\lambda }(\mu )I_{\lambda }(X,\mu ,t)+J_{\lambda }(X,\mu ,t)$$

The source function is defined as increasing radiation intensity per unit thickness in the *μ* direction. Similar to the radiation intensity, the function of wavelength, direction and location can be written as follows [29]:

(3) $$J_{\lambda }(X,\mu ,t)=+K_{\lambda }(\mu )I_{b,\lambda }(T(X,t))+\frac{1}{2}\int_{\mu =-1}^{\mu =1}\sigma_{s\lambda }(\mu )\phi_{\lambda }(\mu )I_{\lambda }(X,\mu ,t)d\mu$$

**Figure 1:**
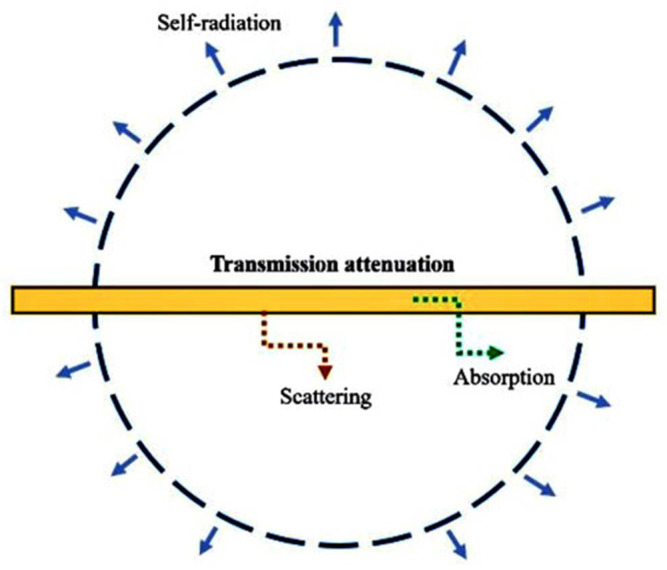
Radiative energy balance in a gas element.

The radiation energy balance terms are schematically presented in Figure 1.

The radiative boundary conditions expressed by integro-differential equations are as follows:

(4) $$\{\begin{array}{c} I_{\lambda }(0,\mu ,t)=I_{\lambda ,b}(f(t))\\ I_{\lambda }(E,\mu ,t)=I_{\lambda ,b}(T_{E}) \end{array}$$

where *E* is the fibrous media length. The monochromatic intensity is expressed by Plank’s law in the situation in which the black body is transparent, given as

(5) $$I(X,T(X,t))=\frac{C_{1}}{\lambda_{5}[\exp(\frac{C_{2}}{\lambda T(X,t)})-1]}$$

where, by experimental analysis, these constants are introduced as *C*_1_ = 1.19 × 10^−16^ ($\frac{W}{m^{2}}$), *C*_2_ = 1.44 × 10^−2^ (m.K). A one-dimensional combined transient energy equation is mentioned as follows [30]:

(6) $$\rho c_{p}\frac{\partial T(X,t)}{\partial t}=\nabla (K\nabla T(X,t)-{\dot{Q}}_{r})+{\dot{q}}_{gen}$$

where ρ is density, *c_p_* specifies heat under constant pressure, *T* is temperature, *t* is time, *K* is thermal conductivity, $\dot{Q_{r}}$ represents the gradient heat flux vector, and the divergence of radiative heat flux in (6) is the net radiant energy emitted from a unit volume. It is a scalar quantity, a function of the position, and is called a radiative source, as presented in Equation (7) [30].

(7) $${\dot{Q}}_{}=2\pi \int_{\lambda =0}^{\infty }\int_{\mu =-1}^{\mu =1}I\left(\lambda ,X\right).\mu \;d\mu \;d\lambda$$

The unknown parameter of the radiation heat equation I(λ.x) is the radiation intensity, which is a function of direction *μ* at a point *X*. The radiation source in one-dimensional heat transfer is given by Equation (8) [31].

(8) $$\nabla {\dot{Q}}_{r}(X)=-\frac{d}{dX}{\dot{Q}}_{r}(X)$$

The following equations are considered to describe radiative conduction heat transfer through a thin fibrous medium with homogeneous properties. The temperature distribution in the x direction, *T(X,t)*, is derived by solving the non-linear equation.

(9) $$\rho c_{p}\frac{\partial T(X,t)}{\partial t}=\frac{\partial }{\partial X}(K(T(X,t))\frac{\partial T(X,t)}{\partial t})+\nabla {\dot{Q}}_{r}$$

The boundary conditions for solving Equation (9) are written as

(10) $$T(0)=T_{0}$$

The conduction heat transfer term is defined as

(11) $${\dot{Q}}_{c}(X,t)=-K(T(X,t))\frac{\partial T(X,t)}{\partial X}$$

The thermal conductivity value ${\dot{Q}}_{c}(X,t)(\frac{w}{m\cdot K}$) is defined as a function of the absolute temperature *KT*.

(12) $$K(T)=K(298.15){\sum_{i}\left(\frac{T}{298.15}\right)}^{t}$$

Moreover, the volumetric specific heat capacity values are defined as a function of absolute temperature *T*, given by Equation (13).

(13) $$\rho c_{p}=(\rho c_{p})(298.15){\sum_{i}\left(\frac{T}{298.15}\right)}^{t}$$

In this problem, the combined heat flux is given by the summation of conduction and radiation. The unknown parameters after coupling Equations (7) and (9) are the monochromatic radiation intensity and temperature field, which are provided by solving Equation (14).

(14) $$\rho c_{p}\frac{\partial T(X,t)}{\partial t}-\frac{\partial }{\partial X}(KT(X,t)\frac{\partial T(X,t)}{\partial t})=S_{r}(X,t)\;\;$$

Boundary conditions for solving the non-linear Equation (14) are written as follows:

(15) $$T(0,t)=f(t)=\{\begin{array}{cc} 50t+300 & 0<t<1\\ 300 & t>1 \end{array}$$

$$T(E,t)=T_{E}$$

$$T(X,0)=T_{E}$$

For solving the transient equations of the layer, assuming that the layer is initially at uniform temperature TE, and also that linear heat shock exists on the initial position of the fibrous medium, the temperature of the ends of the fibrous medium is assumed constant.

### 2.2. Deep Neural Network Method

The essential step in using an artificial neural network algorithm is developing the specific applicable algorithm to find the best correlation between different crucial parameters in a problem. Simulating the problem by discretizing the governing equations, the dataset was imported into the algorithm in order to find the best correlation and output data predictions. The utilized AI algorithm for this research is long short-term memory (LSTM) [31,32]. It is one of the deep neural network (DNN) methods applicable for predicting time series data. The data collected during experiments or simulations are time-dependent. For this reason, LSTM is the most accurate algorithm for predicting the output data of our recent research. For storing the data, the “cell states” are used to store the long-term data in hidden layers. As presented in Equations (16) and (17), *f* and *i* represent the forget and input gates to control the input of each cell [33].

(16) $$f_{t}=g\left(W_{f}\cdot \left[h_{t-1}.X_{t}\right]+b_{f}\right)$$

(17) $$i_{t}=g\left(W_{i}\cdot \left[h_{t-1}.X_{t}\right]+b_{i}\right)$$

In addition, g is a non-linear sigmoid function utilized as an activation function here. Meanwhile, W and b introduce the weight matrix and bias function, $h_{t-1}$ presents the last time step output, and $X_{t}\;$presents the current time step input. 

Moreover, Equation (18) presents the relation to understand the input’s current state,

(18) $${\overset{´}{C}}_{t}=\tanh\left(W_{c}\cdot \left[h_{t-1}.X_{t}\right]+b_{c}\right)$$

where the c index shows each parameter’s current state. Equation (19) uses both the forget and input gates to obtain the current cell state [33].

(19) $$C_{t}=f_{t}*\;C_{t-1}+i_{t}*\;{\overset{´}{C}}_{t}$$

By utilizing the output of each cell state, which is presented as Equation (20), the algorithm output is presented as in Equation (21).

(20) $$O_{t}=g\left(W_{o}\cdot \left[h_{t-1}.X_{t}\right]+b_{o}\right)$$

(21) $$h_{t}=O_{t}*\tanh\left(C_{t}\right)$$

In addition, the o index introduces the cell-state output parameter.

The AI predictions are made using these equations of the LSTM algorithm. Furthermore, for the input’s order reduction, the Mahalanobis distance method is used to reduce the prediction time [34,35]. Then, the simulation is compared with the AI-predicted data to find the fastest and most accurate solution. In a recent study, the best learning rate was 0.01. At this specific number, with low learning rates, the loss improves slowly, and then training accelerates until the learning rate becomes too large and loss increases, so the training process diverges.

**Figure 2:**
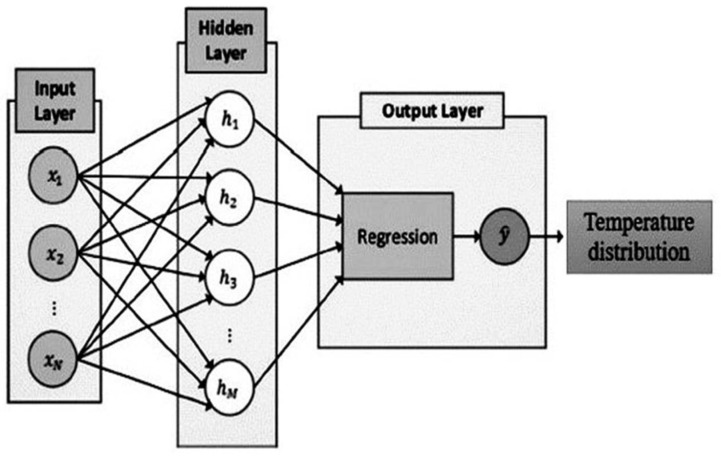
The DNN structure of a recent study.

In this study, the temperature distribution of experimental measurements is predicted with a DNN-based algorithm. As shown in Figure 2, the utilized artificial structure consists of three main layers, named the input layer, hidden layer and output layer.

## 3. Results and Discussion

**Figure 3:**
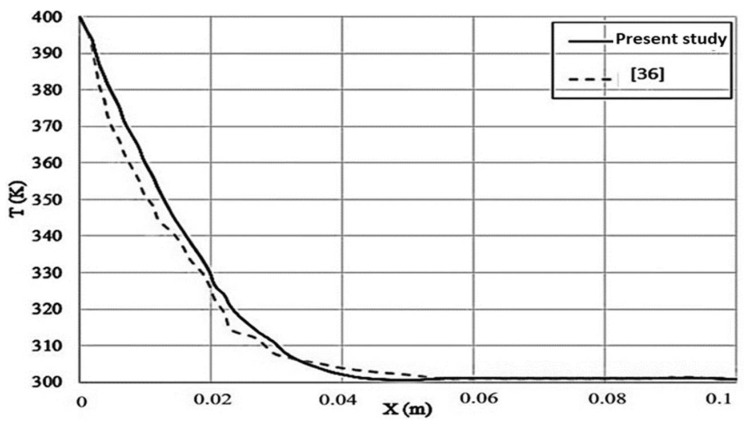
Comparing the results of transient thermal distribution in silica fibers.

This paper focused on the one-dimensional combined heat transfer of PMMA-POF by computer numerical analysis and the LSTM method. The data are validated by Asllanaj et al. [36]. Figure 3 shows the temperature distribution of PMMA-POF during four predictions in different periods and compared with a previous experimental study [36]. Although the source temperature of the front layer of the PMMA sample increases from 300 K to 400 K in 1 s, the external wall temperature remains constant at 300 K.

As presented in Figure 3, the sample’s temperature stands at 400 K in the initial point. Then, it decreases gradually to 300 K in 0.05 m. From the middle point (0.05 m) up to the end point of the sample, its temperature remains constant at 300 K. In detail, the temperature gradient occurs from the beginning of the sample up to 0.05 m approximately. Although this study is verified by that of Asllanaj, the two curves have some differences, which are related to the research method. Moreover, the sample experienced a decreasing temperature trend, similarly to the study of Asllanaj [36]. Moreover, in Asllanaj’s study, the temperature decline rate was sharper than in our study. Two critical points are at the beginning and end of the curves, which coincide. The deviation occurs due to the different approach. Asllanaj used an experimental study in order to determine the decreasing rate of the sample with respect to its length. A limited range of studies were conducted in this special case study. This means that only Asllanaj [36] has performed this experimental research with these particular boundary conditions, this material and combined heat transfer implementations (conductive and radiative). Furthermore, a significant goal of this study was to conduct a comparative analysis between two different schemes (numerical simulation and artificial intelligence methods) with regard to finding the best algorithm among others for this specific case study. Thus, instead of focusing on referencing different studies for validation, we proceed to consider the aims of our own research.

**Figure 4:**
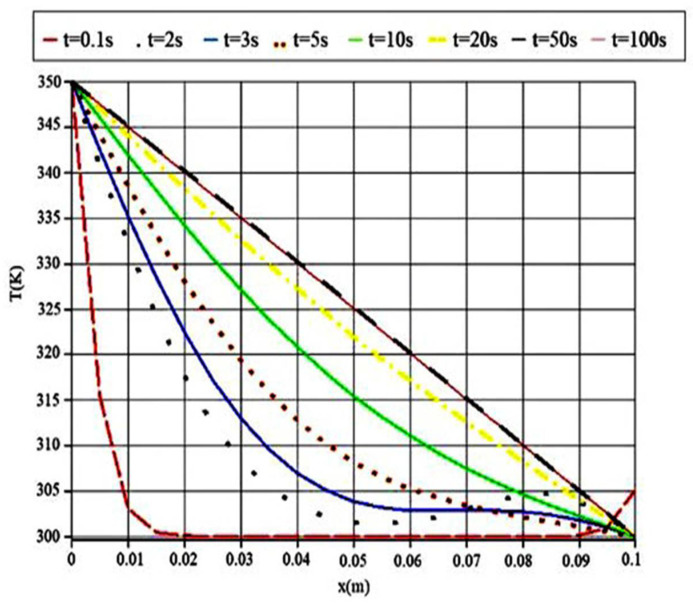
The temperature profile in various time steps.

Figure 4 presents the temperature distribution of PMMA-POF, which was obtained from computer numerical analysis at various times. The utilized temperature values equal f(t) at x = 0 and T_E_ at x = 1.

Figure 4 presents the temperature decreasing rate of the 0.1 m PMMA sample in eight time periods. This investigation was conducted in order to identify the material’s dependencies on the time period. At first glance, it can be understood from the curves’ trend that when increasing the time, the temperature varies slightly in the total length. This means that its gradient is high in the shortest calculation time, and its gradient decreases upon increasing the simulation time. 

Furthermore, for the maximum simulation time (t = 100 s), the decreasing temperature trend along the sample length is entirely constant and the same as the steady condition. As the simulation time decreases, the temperature profile varies and, in the minimum time, it experiences a sudden decrease in the first few lengths. The first and last points of the simulation coincide, but as a result, the temperature curves vary with respect to the simulation time.

**Figure 5:**
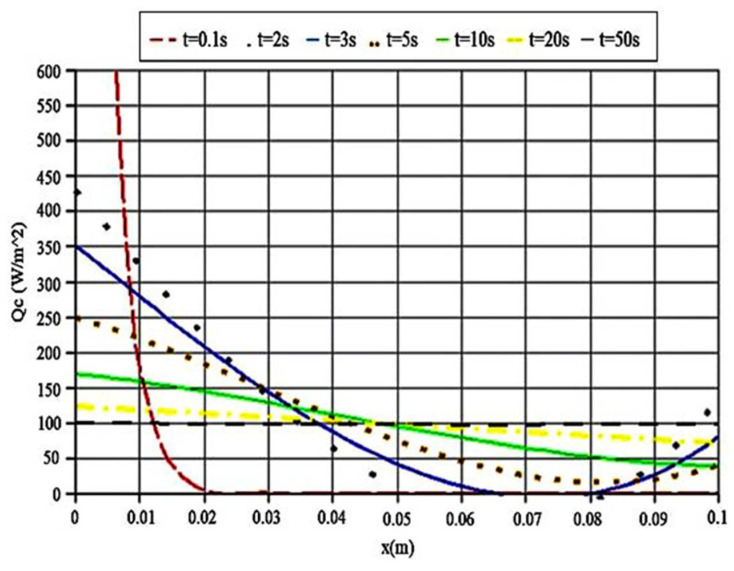
Conductive heat flux in various time steps.

**Figure 6:**
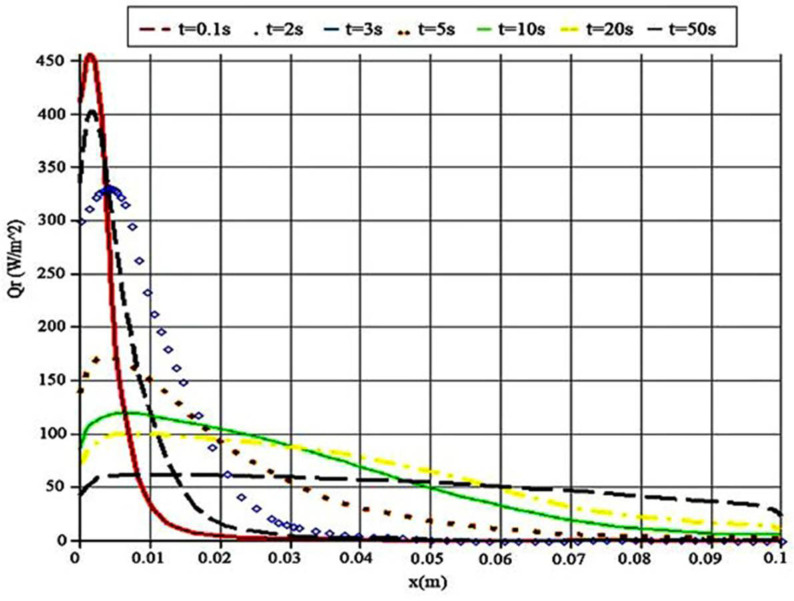
Radiative heat flux in various time steps.

Figure 5 and Figure 6 show the heat flux variations with time and position. The conductive heat flux is similar to the radiative one in the case of behavior. 

As presented in Figure 5, the conductive heat flux at t = 50 converges to stable conditions around 100 ($\frac{W}{m^{2}}$) and it remains stable approximately. This can be considered as the main reason for neglecting t = 50 to t = 100. As a result, durable conductive heat flux in the PMMA sample is obtained with the maximum simulation time. Furthermore, this trend can easily prove the fact that, in order to obtain steady and constant conductive heat transfer along the entire sample, the simulation time should be set at 50 s. When decreasing the simulation time, the output curves do not display a stable trend, and their distances from the stable line increase. The shortest simulation time is t = 0.1 s. It is clear that, at this time, there is a sharply declining trend in temperature in the PMMA sample. Consequently, considering the atomic scale, when decreasing the time, the sample’s atoms do not have enough time to conduct the heat from their previous neighbors to the next ones. This can be considered as a significant reason that there is a noticeable temperature fall in a short time of simulation.

As presented in Figure 6, the most stable condition occurs with the maximum simulation time when the radiative heat transfer reaches 50 ($\frac{W}{m^{2}}$) approximately at 0.06 m. The radiative heat transfer cannot be the same as conductive heat transfer, but it also displays some slight decreasing trends. The basic rule is related to the simulation time, as shown in Figure 6, in the case of increasing time, but when these two figures are compared with each other, the amount of conductive heat transfer is found to be twice the radiative heat transfer. As a matter of fact, in the same physical conditions, conduction plays a vital role in heat transfer from PMMA samples.

**Figure 7:**
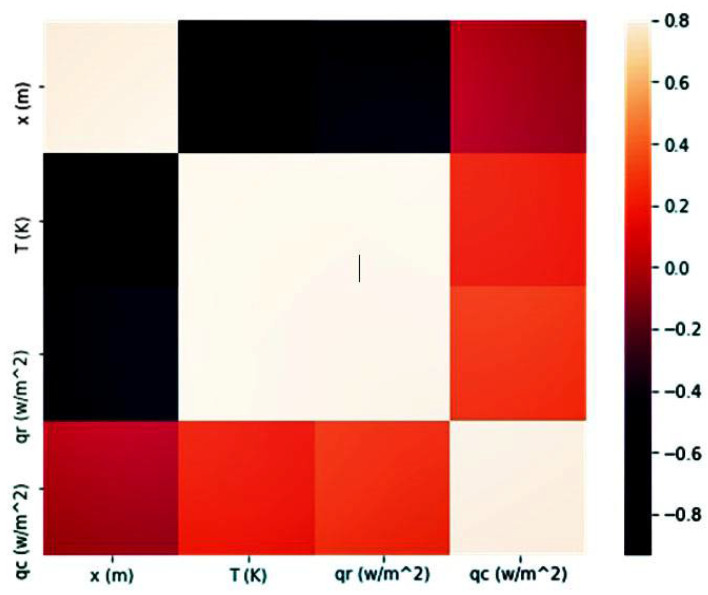
The correlation matrix between parameters.

However, when these solutions are obtained by an artificial intelligence method, the datasets have fewer data than expected in the case of experimental data. We considered testing time (s), temperature distribution (K), radiative heat transfer ($\frac{W}{m^{2}})$ and conductive heat transfer ($\frac{W}{m^{2}}$). The parameters’ correlation is expressed as a value between 0 and 1. A correlation matrix should be generated to visualize the most correlated parameters through Pearson’s correlation method, as shown in Figure 7. This method utilizes a heat-map visualization in order to identify the best relation between different parameters. It presents the values with varying spectra of dark and light colors. In this spectrum, the lighter one shows the best relation. Hence, it is obvious that we can find a good relationship between important parameters by using the AI prediction method.

As can be inferred from Figure 7, the correlation matrix clarifies the relations of different parameters, which are predicted by the LSTM method. This type of presentation helps us to understand the intensity of these relations better. The darkest color presents the least related parameters, and the brightest color indicates the most related ones. The most relevant parameters are temperature and radiative heat transfer. Furthermore, the least relevant ones are the relation between sample length and temperature distribution, and the other one predicted by the LSTM method is the combination of radiative heat transfer and sample length.

**Figure 8:**
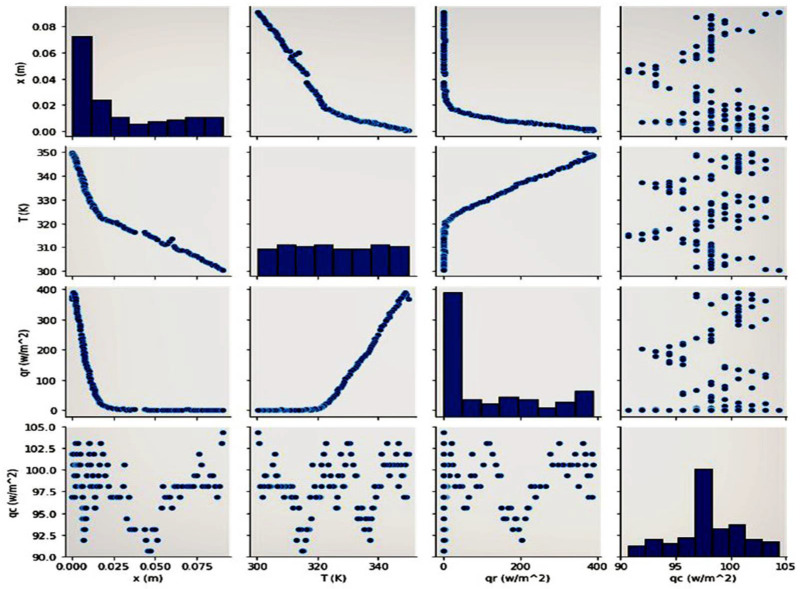
The scatter plot of different heat transfer parameters.

**Figure 9:**
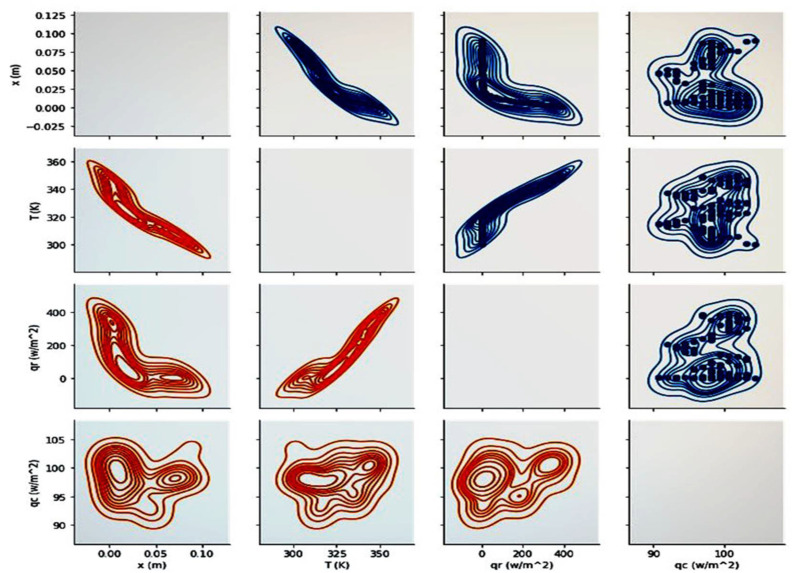
The overall figures of different parameters’ correlations.

Pearson’s correlation method is used to find the relation between all of the effective parameters of this study. Moreover, the LSTM-predicted parameters are plotted as a matrix of figures. Hence, we can find that the numerical solution in this problem can measure positive magnitudes. Despite this, our utilized method predicts both negative and positive values simultaneously. Moreover, Figure 8 presents the linear regression between the correlation’s scatter data. This figure is the same as Figure 9, but the data showed the best fit. 

One of the most significant advantages of using an AI-based method is that it is independent of the type of the problem, in the fields of both physics and chemistry. Thus, this investigation can be developed for different boundary conditions and types of samples. However, due to the nature of the present prediction, regression is utilized in order to predict different parameters. For instance, the conductive heat transfer of different sample lengths can be predicted easily by following the proposed method. Consequently, other conditions can be estimated by the previously suggested algorithms.

Figure 8 shows the predicted values as a correlation matrix of different variables at t = 50 s. The results show that when moving along the PMMA sample, the conduction and radiative heat transfer rates decrease and reach a constant value. Fixing these values leads to a decrease in temperature distribution. Each element of these rows and columns represents a particular relation between the effective parameters if it is imagined that the basic cross-line presents the relation of the same parameter. Moving upward from the cross-line indicates the reverse connection of each downward element. The x-axis was replaced with the y-axis in a reverse procedure to obtain more details.

Furthermore, Figure 9 presents a gradient descent of different parameters obtained by the cost function optimization. The gradient descent presents the possible answers. The compressed lines indicate the nearest solution among others. Visualized as a slope, the compressed lines have the lowest value of gradient descent and scattered ones have the least amount of gradient descent. These charts, especially those in Figure 9, demonstrate how the subset of scattering solutions can be optimized to find the best-fitting ones.

**Figure 10:**
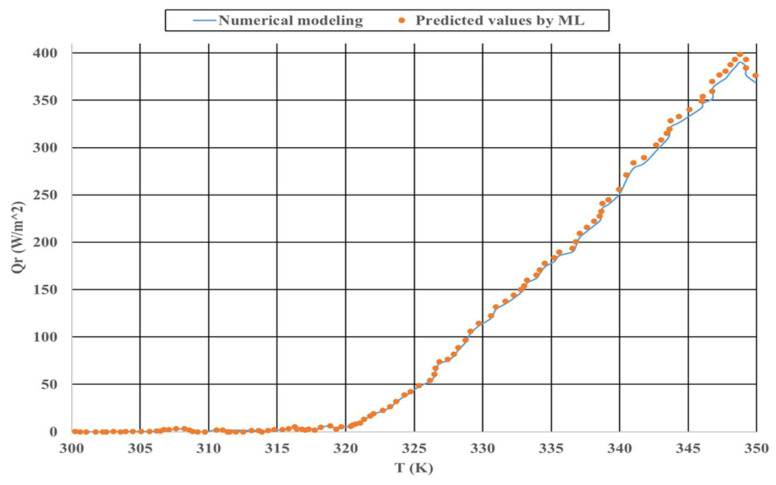
Comparison between the two utilized different methods.

The comparison between numerical and experimental modeling is presented in Figure 3. The machine learning method was utilized, and it was compared with numerical methods to determine the best approach in this study. Figure 10 presents this comparison.

As presented in Figure 10, the predicted values coincide with the numerical method in most of the points. However, some neglectable differences are observed; these are related to selecting a hyperparameter for the LSTM algorithm.

**Figure 11:**
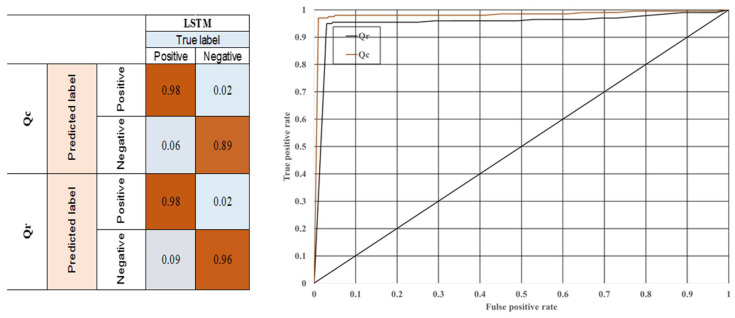
ROC curve and confusion matrix of radiative and conductive heat transfer prediction.

One of the vital tasks in machine learning implementation is to evaluate the utilized algorithm with the ROC curve and confusion matrix (Figure 11). Furthermore, another important task is to examine the accuracy of their performance. The algorithms utilized for estimating the conductive and radiative heat transfer were assessed, and the models’ performance is reported in Table 2. Statistical analysis was performed and evaluated using MAE, root mean square error (RMSE), accuracy (ACC), specificity (FPR), sensitivity (TPR), positive predicted values (PPV) and true negative rate (TNR).

As presented in Figure 11 and Table 1, the confusion matrix shows whether the LSTM algorithm is accurate enough to predict different heat transfer problem values. As is demonstrated, the accuracy for both parameter predictions is 0.98. Consequently, this algorithm’s accuracy is strong proof of the advantages of using machine learning methods instead of traditional expensive and slow computations. At first glance, it is evident that the LSTM algorithm, with the proposed accuracy, can predict these parameters more efficiently while decreasing the calculation time and cost.

## 4. Conclusions

A new comparative artificial intelligence prediction method for the heat transfer modeling of PMMA samples based on coupled heat transfer is proposed and validated with other works. The analysis of the combined heat transfer of a PMMA sample as the core of a fibrous medium is solved by the transient coupled system of equations. This is achieved by computer implementation with the implicit finite difference scheme for conduction and the Gaussian numerical method for the radiation heat flux. The temperature distribution is validated with another experimental study, and its deviation is caused by different schemes. The results present an intense gradient of the temperature values in the primary positions, indicating the spectral amount of conductive heat flux and the heat flux of the two heat transfer types. Hence, the solution depends on the temperature distribution becoming a steady state at the same time. It can be clarified that the changing trend of the sample temperature with respect to its length varies when increasing the total time in the simulation. Consequently, this phenomenon can be attributed to the atomic structure of the PMMA. By increasing the time, heat can be conducted to the next atoms easily. Meanwhile, in t = 100 s, the total temperature varies slightly in the sample’s length. On the other hand, by decreasing the total time, there is a sharp temperature decrease in the first atomic regions of a sample. It is proven that the conductive and radiative heat flux are similar in the case of trending behavior, but it is two times higher, approximately. Furthermore, their total flux becomes approximately constant at the steady-state condition. In our research, the artificial intelligence method and numerical results demonstrate good agreement and verify the novel LSTM algorithm. The overall calculation time is increased to 2 s by using this method. As indicated in this study, the LSTM method can easily predict and evaluate these types of problems in different conditions, and it is also the most accurate one (98%), which is verified by the confusion matrix and ROC curve. This method can predict the temperature distribution in a PMMA sample. It will help to find the best industrial results in less time. It improves the prediction speed when compared with numerical solutions. In further investigations, the proposed LSTM algorithm can be an effective and fast tool to predict a PMMA sample’s most effective parameters regarding its combined heat transfer. To be more specific, industrial applications can add this module when implementing their performance tests and predict an approximate value of heat transfer for their sample, whether conductive or radiative heat transfer. This will allow research groups to select the best design for their proposed application.

## Figures and Tables

**Table 1:** Material properties of PMMA.

Property	Value
Density (g/cm^3^)	1.18
Surface Hardness	RM92
Tensile Strength (MPa)	70
Flexural Modulus (GPa)	2.9
Linear Expansion (/°C × 10^−5^)	7
Max. Operating Temp. (°C)	50

**Table 2:** The learning accuracy and its performance as evaluated by different measurement tools.

Parameter	Method	TNR	PPV	TPR	FPR	ACC	RMSE	MAE
Qc	LSTM	0.98	0.98	0.94	0.02	0.96	16.42	0.06
Qr	LSTM	0.98	0.98	0.92	0.02	0.95	37.53	0.07

## Data Availability

The data available through corresponding author.

## Nomenclature

**Parameter**	**Description**	**Parameter**	**Description**
J	Monochromic radiation intensity	${\dot{Q}}_{r}$	Radiative heat flux
λ	wave length	${\dot{q}}_{gen}$	Heat source flux
t	time	I	Radiation intensity
X	direction	E	Fibrous media length
μ	direction	f	forget gate
c	propagation speed	i	input gate
ρ	density	g	Non-linear sigmoid function
$c_{p}$	Specific heat	O	cell-state output parameter
T	Temperature	W	Weight function
K	thermal conductivity	$X_{t}$	Recent gate information
